# Temporal ghost imaging for pump–probe X-ray solution scattering

**DOI:** 10.1107/S2053273325004243

**Published:** 2025-07-07

**Authors:** B. R. Mobley, Kevin E. Schmidt, R. A. Kirian

**Affiliations:** a Department of Physics Arizona State University,Tempe AZ 85287 USA; Deutsches Electronen-Synchrotron, Germany

**Keywords:** SAXS, small-angle X-ray scattering, ghost imaging, Bayesian, XFELs, X-ray free electron lasers, pump–probe

## Abstract

Time-resolved small- and wide-angle X-ray scattering is a valuable tool for investigating biomolecular dynamics on a wide variety of timescales, without cryo-freezing or crystallization. However, some systems, such as the initial excitation of photo-active proteins, evolve dynamically on timescales that may be faster than the duration of the pump and probe beams. Data from a single pump–probe pulse pair therefore contain information from a mixture of time points. In this work, a simple algorithm is developed to recover the dynamics of solution scattering profiles. It leverages information about the pump and probe pulse beams’ temporal profiles by using the same mathematical framework as ghost imaging [Pittman *et al.* (1995). *Phys. Rev. A***52**, R3429–R3432; Bennink *et al.* (2002). *Phys. Rev. Lett.***89**, 113601; Gatti *et al.* (2004). *Phys. Rev. Lett.***93**, 093602]. Results from several simulated data sets are presented.

## Abstract 2

An algorithm is described to recover super-temporal resolution dynamics of X-ray scattering profiles of photo-excited biomolecules in solution from pump–probe X-ray solution scattering data.

## 1. Introduction

Time-resolved small- and wide-angle X-ray solution scattering has been used to study a variety of systems on a wide range of timescales. The techniques can be used to study solutions undergoing several kinds of perturbations such as a temperature jump (Thompson *et al.*, 2019 ▸), pH jump (Rimmerman *et al.*, 2019 ▸) or photo-excitation (Cho *et al.*, 2010 ▸; Arnlund *et al.*, 2014 ▸; Levantino *et al.*, 2015 ▸; Lee *et al.*, 2021 ▸; Schotte *et al.*, 2024 ▸). The data collected do not provide sufficient information to retrieve the full, three-dimensional structure of the molecules in solution, but nonetheless provide valuable structural information. Ultrafast time-resolved, three-dimensional structural information can be prohibitively costly to acquire at many time points via serial femtosecond crystallography (Chapman *et al.*, 2011 ▸; Barends *et al.*, 2022 ▸), so the time-resolved structural information from solution scattering studies can supplement information between these time points. In addition, many dynamical processes in biomolecules cause conformational changes that cannot be supported by the crystal lattice in crystallographic studies; thus solution scattering is often the only way to study these dynamics (Neutze, 2014 ▸; Konold *et al.*, 2020 ▸).

In this work, we will focus on using these techniques to study some of the fastest processes in biology, the evolution of photo-excited targets. These are processes such as the conformational changes in rhodopsin following photo-absorption, which form the molecular basis of vision, or in the photosystem complexes, the molecular basis of photosynthesis. The pump–probe approach used to study these targets involves scanning the time delay between an optical pump pulse, tuned to the absorption of the target, and an X-ray probe pulse, which yields diffraction measurements that evolve as a function of the delay. In this scheme, the timescale of the evolution of the molecules is usually assumed to be significantly larger than the combined temporal width of the pump and probe pulses in order to observe snapshots of the dynamics akin to stroboscopic imaging. The usage of the extremely short duration pulses of X-ray free electron lasers (XFELs) for the probe allows for the interrogation of rapid processes, but there are still many processes with temporal dynamics on the order of the combined timescale of the pump and probe pulses in these experiments which cannot be accurately measured in this straightforward way. The dynamics following photo-absorption tend to evolve according to logarithmic timescales that can span more than ten orders of magnitude from photo-absorption to signal transduction (Standfuss, 2019 ▸; Wickstrand *et al.*, 2019 ▸). Later stages of the dynamics can be resolved to sufficient resolution by modeling the signals as a mixture of just a few states (Meisburger *et al.*, 2021 ▸), but the initial, ultrafast processes of photo-absorption are obscured by the temporal widths of the pump and probe beams. To study these ultrafast processes, we can leverage knowledge of the pump and probe pulse profiles in order to reconstruct the super-resolution dynamics – that is, dynamics on timescales shorter than the duration of the pump and/or probe pulses. The computational framework known as ghost imaging (Pittman *et al.*, 1995 ▸; Bennink *et al.*, 2002 ▸; Gatti *et al.*, 2004 ▸) applied in the time domain (Ryczkowski *et al.*, 2016 ▸) uses measurements of the beam pulses, whether they are detailed individual pulse measurements provided by an instrument such as the X-band Transverse Deflecting Mode Cavity Instrument (XTCAV) (Dolgashev *et al.*, 2022 ▸; Ren *et al.*, 2020 ▸) at the Linac Coherent Light Source (LCLS), or bulk measurements of the statistics of the beam, to compute the reconstruction. Similar algorithms have been applied to X-ray absorption data in the frequency domain (Li *et al.*, 2021 ▸), to single self-amplified spontaneous emission (SASE) pulses acting simultaneously as both pump and probe (Ratner *et al.*, 2019 ▸), and even to the problem of characterizing the SASE pulse itself (Li *et al.*, 2022 ▸). For the case of solution scattering of evolving mixtures, an algorithm called REGALS (Meisburger *et al.*, 2021 ▸) uses a similar procedure in each step of its iterative architecture to recover both the unknown component mixture scattering profiles and their unknown time evolution. In this work, we will focus on the recovery of super-resolution dynamics of the solution scattering profiles of biological targets. This will allow X-ray sources with cutting-edge pulse duration to study the initial ultrafast dynamics of photo-absorption as well as allow sources with longer pulse duration such as the Compact X-ray Light Source (CXLS) at Arizona State University (Graves *et al.*, 2014 ▸) to extend the range of timescales they are able to study.

## 2. Theory

We wish to reconstruct the solution scattering profile of a molecule species as a function of scattering magnitude and time from diffraction data that carry information about many molecules at different pump–probe time delays incoherently summed on the detector. We will call the temporal scattering profile of the molecule species and its associated solvent cage 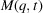, where *t* represents time and 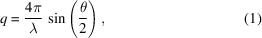
where θ is the angle between the incoming and scattered light. If we assume the dynamics of the molecules in solution are driven by single-photon absorption and are independent from one another, we can model the dynamics of the solution as individual molecules, each absorbing a photon at random times during the pump pulse with the probability of an absorption event proportional to the strength of the pump pulse at that time. Let us assume that each molecule, once excited, then proceeds along the same dynamic trajectory, resulting in a mixture of molecules at different points along this trajectory. In a real ultrafast pump–probe experiment, the scattering from excited molecules will initially not be isotropic due to the preferred excitation of molecules with transition dipole moments aligned with the polarization of the laser among other effects (Choi *et al.*, 2022 ▸). For simplicity, we will ignore this anisotropy in the present work, though the technique presented here can easily be generalized to account for it. The scattering profile of our mixture will thus be an incoherent sum of the scattering from molecules at random orientations and at different stages of identical dynamics. Not all of the molecules will be excited, though, so the total solution scattering profile will also contain a static component of the molecules’ dark state as well as solvent scattering, which we will also assume is static. This gives us 
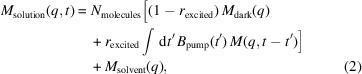
where 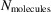 is the number of target molecules in solution, 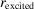 is the ratio of the target molecules that are excited by the pump, 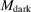 is the scattering profile of the unexcited target molecule, 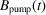 is the probe pulse’s temporal profile and 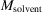 is the scattering profile of the solvent. We have chosen to define the pump profile in a normalized way, 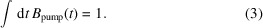
The trajectory of the excitation of the molecules must start from the dark state, so let us define 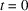 as the moment of excitation and assume 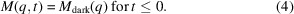
This construction ensures that the full solution scattering will be the sum of the dark state and solvent at all times before any portion of the pump pulse has arrived.

The probe pulse will measure an incoherent sum of the evolving mixture’s scattering intensities at many different time points distributed according to the probe pulse’s temporal distribution. We can write this as 
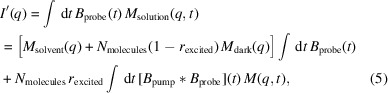
where 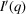 is the scattering density, 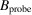 is the probe pulse temporal profile, and 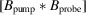 is defined as the following integration:
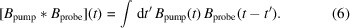
We can rewrite this as 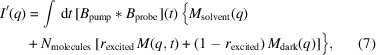
according to our convention of normalizing the probe pulse. This gives us the continuous scattering density at any point on the detector, but, in our detections, this scattering density is integrated over the surface of the pixels with the polarization factor to give the expected intensity, which we will call *I*.

If we assume that the pixels are significantly smaller than the scale of the variations in the intensity, we can approximate the integrated incident scattering density on a pixel as the solid angle spanned by that pixel multiplied by the scattering density and polarization factor at its center. We have assumed our solution scattering patterns will be purely radial, so we can construct annular composite pixels by summing the readout of the included small rectangular pixels. Our measurements are thus samples of the expected intensity on a radial grid. This aggregating of pixels is only for the convenience of boosting signal and not fundamental to the technique. If the scattering signals are anisotropic and the signal strong enough in each pixel, each pixel could instead be considered separately. To proceed with calculations, we will also employ a numerical grid in time to discretize our integral. That is 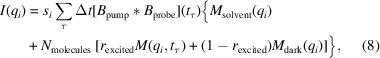
where 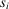 is the solid angle of the *i*th annular composite pixel, 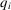 is its radius in *q* space, 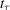 is time of the τ-th time bin and 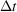 is the size of our time bins. We will assume that 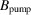 is the same for every measurement. Its center will be our definition of the origin of the time axis, and we will assume it is Gaussian with a known width and negligible shot-wise fluctuations. These assumptions are only for simplicity. It is entirely possible to use diversely structured pump pulses, which has been previously achieved in crystallography for a particular set of temporally structured pump pulses in the HATRX technique (Yorke *et al.*, 2014 ▸). Here, we will focus on the structure of the SASE pulses and thus limit ourselves to these simple reproducible pump pulses. We will assume 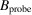 is finite in duration, at least approximately Gaussian, and will vary, especially in the time it is centered around as we experimentally scan the delay between pump and probe pulses.

If we measure the intensity with many pump–probe temporal profiles, we get an equation of the form 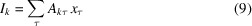
for each composite pixel where 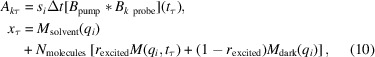
and *k* indexes the *k*th shot. We could use an additional index to track the annular pixels but, as all of the pixels are independent in our formulation, we will drop this index for notational convenience and only discuss the operations necessary for a single composite pixel, keeping in mind that similar calculations are performed serially or in parallel for all other pixels. We will call the *k*th measurement of the composite pixel’s intensity 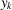. Due to the noise inherent in the scattering of photons and in the detector readout, this will be a sample of some probability distribution with mean 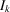.

If we approximately know at what time delay the *k*th probe pulse arrives, which we will call 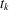, we can make the naive assumption that the pump and probe pulses are very brief compared with the timescale of the target dynamics. We can thus make a naive reconstruction of the temporal profile by binning our measurements into our time grid, and averaging measurements that fall in the same bin. That is 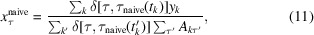
where 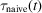 is a function that returns the index of the time bin that the time *t* falls within, and 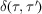 is the Kronecker delta between τ and 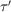. This reconstruction will be blurred by the average combined width of the pump and probe pulses.

### 2.1. Temporal ghost imaging, noiseless case

In the theoretical case that we can measure the intensity without noise or timing jitter, all we need is to make *N* measurements at each pixel with linearly independent combined pump–probe beam profiles, where *N* is the number of time bins we are using to describe our time dimension. This makes *A* an invertible square matrix, whose inverse will reconstruct the temporal profile,
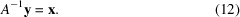
If we measure the pixel intensities more than *N* times, *A* will no longer be a square matrix, and the beam profiles will no longer be linearly independent. In this case, we can solve for 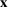 by using the pseudo-inverse, denoted here by the superscript ‘pinv’, 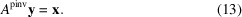
The pseudo-inverse is the singular value decomposition analog to a square matrix inverse. The singular value decomposition of *A* is 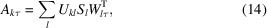
where *U* and *W* are unitary matrices of different sizes, and 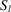 is the *l*th nonzero singular value, all of which are positive. The pseudo-inverse is 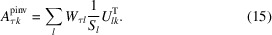
This takes care of the linear dependencies and solves for 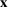 exactly.

### 2.2. Bayesian formulation of Gaussian noise case

Now let us consider the case that the pixel measurements are the intensities with some Gaussian noise added to them. We have 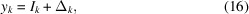
where 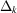 is sampled from a Gaussian distribution with width σ and mean zero. If we assume all of our pixel detections are independent, the joint probability of our measurements, 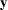, given the expected intensities, 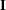, is 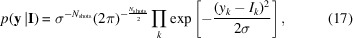
where 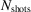 is the number of shots in the data set. To reconstruct the best 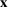 given our data, we write the likelihood in terms of *A* and 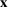, 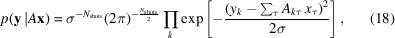
and find its maximum with respect to 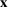 by differentiating the log of the likelihood. Logarithms are monotonically increasing functions; thus the argument of the maximum of the log likelihood is the same as that of the unaltered likelihood. Maximizing this log likelihood is equivalent in this Gaussian noise case to minimizing the square differences of the data and their expected values. We have 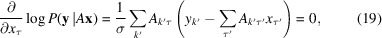
the solution of which is the same as equation (15). This solution suffers when the matrix *A* has any very small singular values, which most matrices do. These small singular values are a problem because the pseudo-inverse will have very large singular values in those directions. When we perform the reconstruction 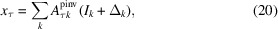
the noise will have randomly sized components in the enormously amplified directions, resulting in the reconstructions, though technically optimal, being entirely unphysical. We can combat the unphysical reconstructions by using a prior distribution to restrain our reconstructions to what we consider physically reasonable, as elaborated in the next section.

#### 2.2.1. Gaussian priors

From the Bayesian viewpoint, we can include in our objective function a prior distribution on **x**, which, when normalized, turns our function into the probability distribution of possible temporal profiles, the posterior. When maximizing the log posterior, we can see that the addition of a prior term is a form of the process often encountered in optimization problems known as regularization, where one adds a term to the objective function to make an otherwise degenerate solution unique. We can make many choices for this prior. We choose to use only Gaussian priors because they are conjugate to our Gaussian likelihood, that is, they result in a Gaussian posterior. The general Gaussian prior on **x** is 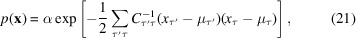
where α is the normalization, 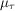 is the expected temporal profile value at the τ-th time bin and *C* is the covariance matrix of the temporal profile values over all the time points. Our optimal reconstruction is now the argument of the maximum of the log posterior. The corresponding extremum condition is 
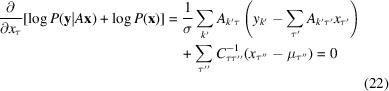
for all τ with the solution 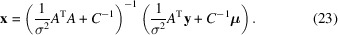


A simple choice of prior is to consider very large magnitude temporal profile values less likely as a simple way to avoid the explosion caused by the large singular values of 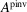. We can implement this consideration by using an uncorrelated spherical Gaussian prior, that is, 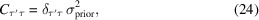
where δ is the Kronecker delta, and 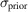 is the magnitude scale within which we expect the temporal profile to fall. Our prior distribution on **x** is thus 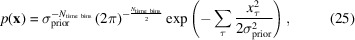
where 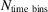 is the number of time points we are using to describe the temporal profile. This prior, together with our likelihood, has the maximum posterior solution 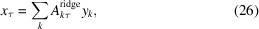
where we define 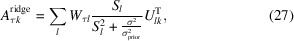
with *U*, *S* and *W* defined as in equation (14). The label ‘ridge’ is derived from Ridge regression, a regularization technique that corresponds to this choice of prior.

This new matrix is like the pseudo-inverse but with a smooth cutoff in the singular values, suppressing the large singular values that resulted in the runaway amplification of noise in the reconstruction. It is equivalent to the pseudo-inverse in the limit that 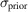 is very large, corresponding to a uniform prior on the temporal profile magnitude. This prior has the problem that, in any regions where there is insufficient information, it will drag the reconstruction to zero and introduce edge effects on the order of the magnitude of the temporal profile. To improve this, we can center our prior on our naive reconstruction. That is 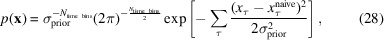
where 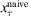 is the value of the naive reconstruction at the τ-th time bin. Our reconstruction, equation (23), in this case becomes 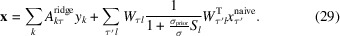
There are many other available choices for Gaussian priors. For example, we could include in our covariance matrix off-diagonal components designed to favor temporal profiles with some measure of continuity. We did not find these to be necessary to achieve a good reconstruction.

## 3. Simulated data

**Figure 1:**
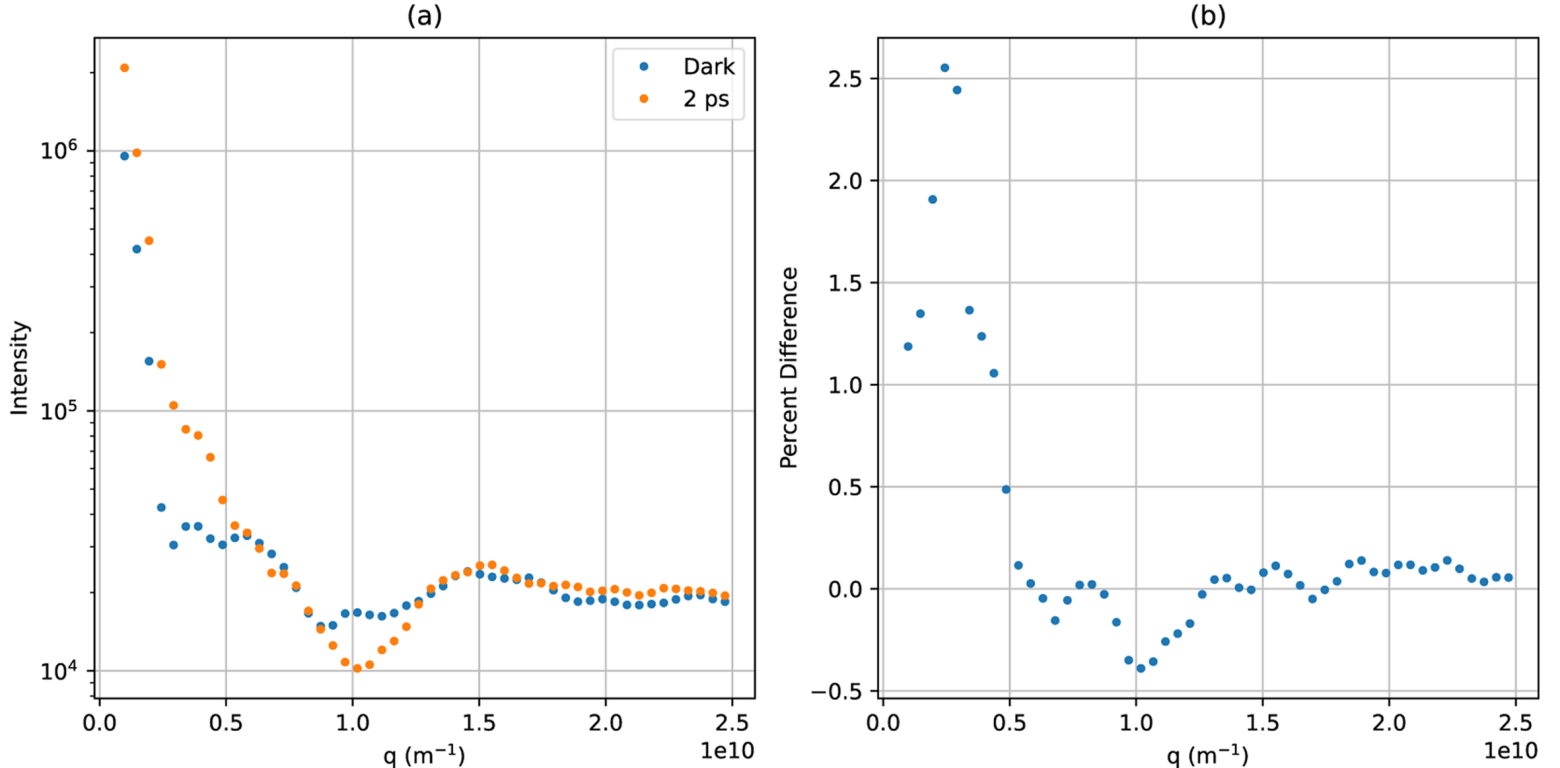
Pictured in (*a*) is the scattering profile of the toy calmodulin model. Pictured in (*b*) is the per cent difference of the final state compared with the dark state.

**Figure 2:**
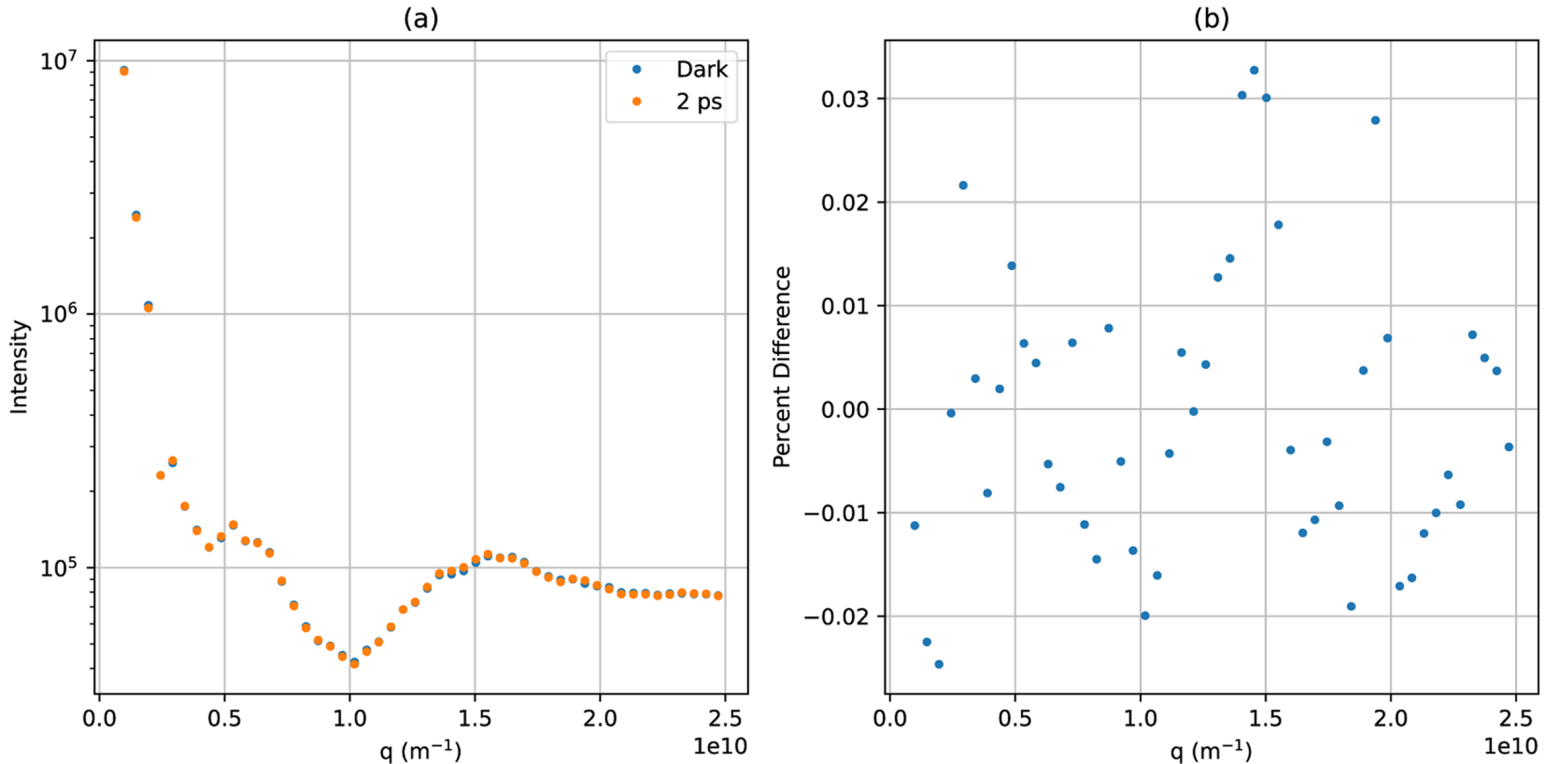
Pictured in (*a*) is the scattering profile of the rhodopsin model. Pictured in (*b*) is the percentage difference of the final state compared with the dark state.

To simulate photon-count diffraction data, we sampled Poissonian noise for each annular composite pixel with mean parameter equal to the expected intensity incident on it, as calculated by equation (8). For simplicity, we assumed no solvent background scatter and that all of the target proteins were excited by the pump, that is 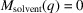 and 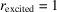. We simulated two temporal profiles of the target protein–water contrast and hydration layer solution scattering profiles by employing the *DENSS* software package (Grant, 2023 ▸). The first temporal profile is a toy model designed only to demonstrate the algorithm. It is based on the solved structure of calmodulin in its calcium-bound (Chattopadhyaya *et al.*, 1992 ▸) and calcium-free (Kuboniwa *et al.*, 1995 ▸) states. We chose this molecule for its large conformational change which results in a significant difference between the initial and final state scattering profiles, as shown in Fig. 1 ▸. This conformational change is brought about by binding to calcium ions and not photo-excitation, but we will take this conformation change in order to generate a temporal profile to test the algorithm. The dynamical evolution for this toy model is a simple interpolation between the simulated solution scattering profiles of these two states at a ratio that changes with some function that has temporal features at many timescales and tails that asymptotically approach solutions containing only the calcium-free or the calcium-bound state. Our second temporal profile is a more realistic model designed to test this method’s feasibility in real experiments. It is based on the solved structures for rhodopsin in its dark state and in its photo-excited state 1 ps after photo-excitation (Gruhl *et al.*, 2023 ▸). The conformational change between these two structures is very subtle, as seen in Fig. 2 ▸. The dynamical evolution for this temporal profile is again a simple interpolation between the two states, this time with the ratio evolving as a logistic curve with a timescale of 100 fs. The temporal profiles and all of the discrete time calculations were represented with 100 time points with discrete time as the ground truth.

**Figure 3:**
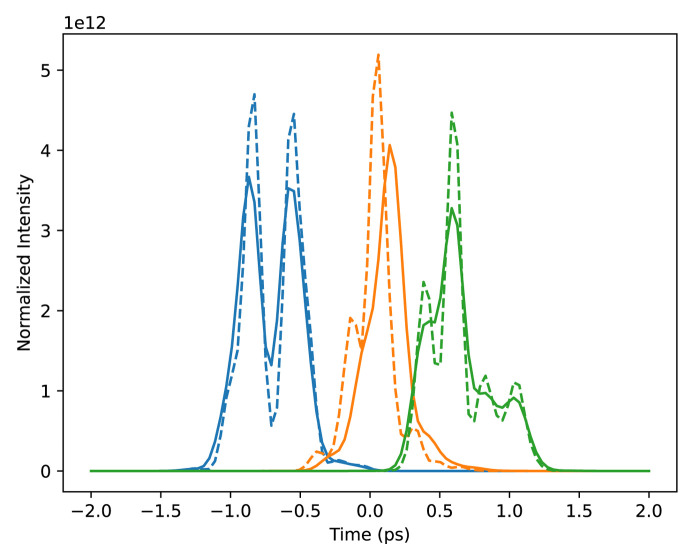
Plotted with solid lines are three samples of the distribution we designed to roughly simulate the temporal profile of a SASE pulse. Plotted with dashed lines are corresponding blurred and randomly shifted versions of each of the pulses that loosely represent a measurement of the pulse with an XTCAV. The average temporal width of the underlying distribution is 500 fs. The scales of the blurring and random shifts are both 50 fs.

**Figure 4:**
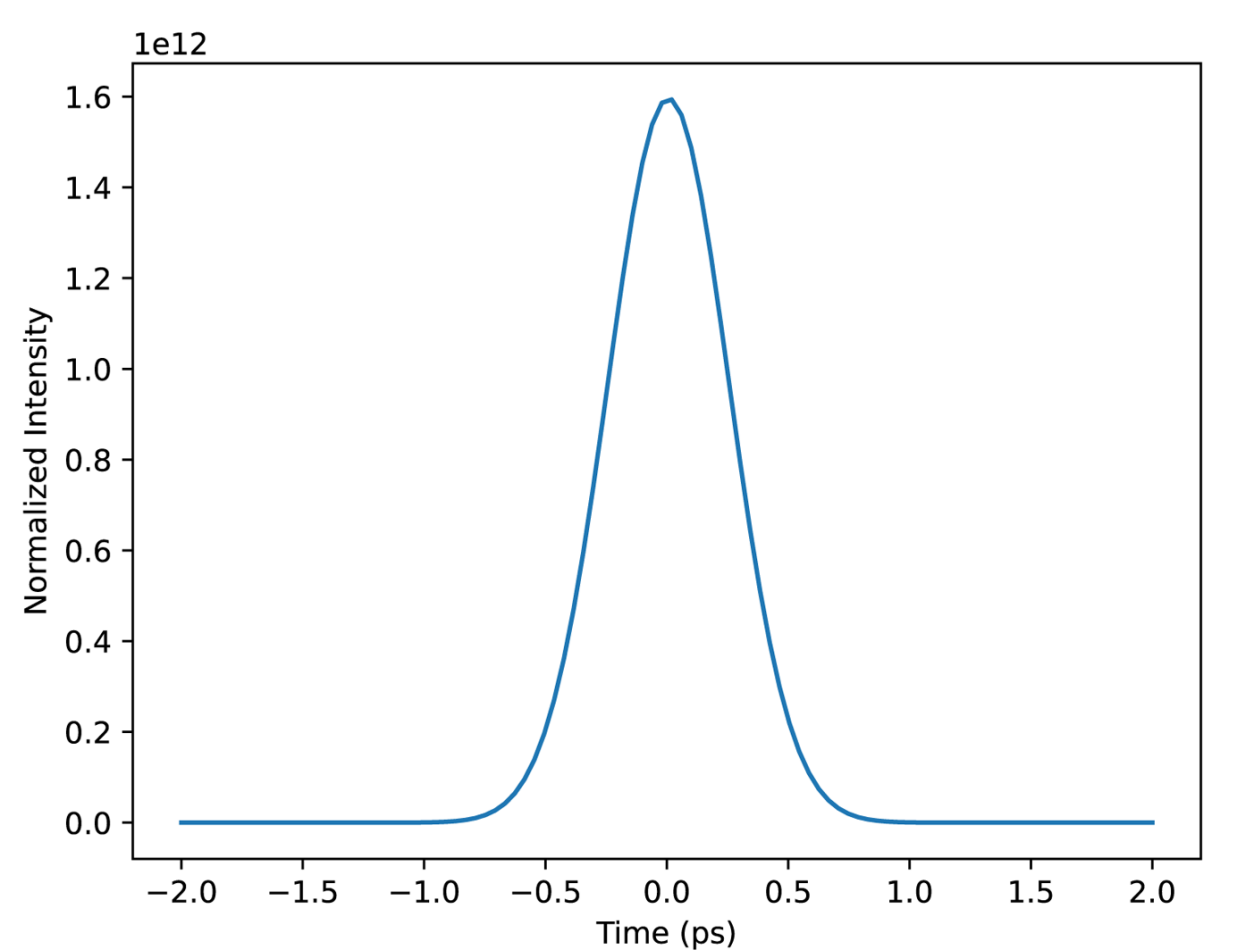
The pictured Gaussian is the limit of many averaged single-shot temporal distributions. Its width is 600 fs, the combined temporal width of the pump, probe and temporal jitter.

For each of these temporal profiles, we simulated two data sets using two different considerations for the combined pump–probe temporal profile. The first loosely models the temporal profile of a Gaussian pump pulse, which we assume is well characterized and perfectly reproducible, and an individual XFEL pulse, which is roughly Gaussian but spikey in a random way due to the process of SASE and includes some level of time jitter (*i.e.* shot-wise variations in the pulses’ centroid times). We simulated the pump–probe beam profiles by sampling random amplified noise modulated by a Gaussian envelope, then blurring them with a Gaussian convolution. In addition, we loosely simulated measurements of the XFEL pulses by an XTCAV-like instrument by convolving the underlying pulses with another blurring Gaussian, then shifting them in time by a random jitter sampled from a zero-centered Gaussian with the temporal jitter timescale as its standard deviation. This way, we have a measurement of the beam matrix that is slightly off from the true matrix. Examples of single-shot SASE and XTCAV temporal profiles are shown in Fig. 3 ▸. The second consideration of the pump–probe temporal profile models the result of many of these SASE probe beams added together according to the central-limit theorem. In this case, the beam temporal profile has a Gaussian distribution with width equal to the width of the lumpy SASE pulse timescale and jitter timescale added together. Using this beam simulates taking many measurements at the same delay and adding the resulting photon counts. This ensures that we know the beam matrix very well even without shot-wise measurements by averaging out the shot-wise fluctuations. An example of this beam profile is shown in Fig. 4 ▸.

**Figure 5:**
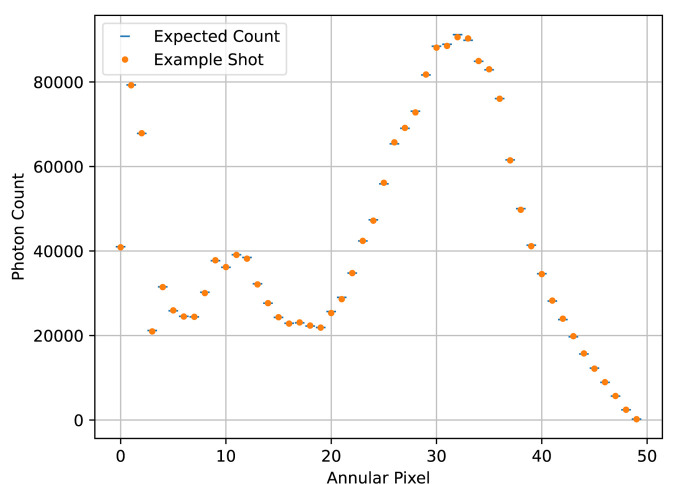
Pictured is an example of a single shot in the rhodopsin data set. The difference in shape, log scale considered, compared with Fig. 2 ▸ is due to the different angular sizes of the annular pixels.

**Figure 6:**
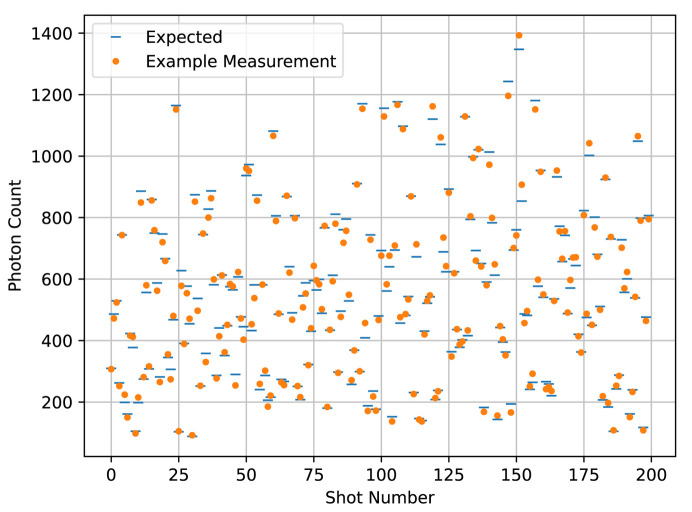
Pictured is an example of photon-count detections from one shot for each of the 200 time delays as detected by the 25th annular pixel.

**Figure 7:**
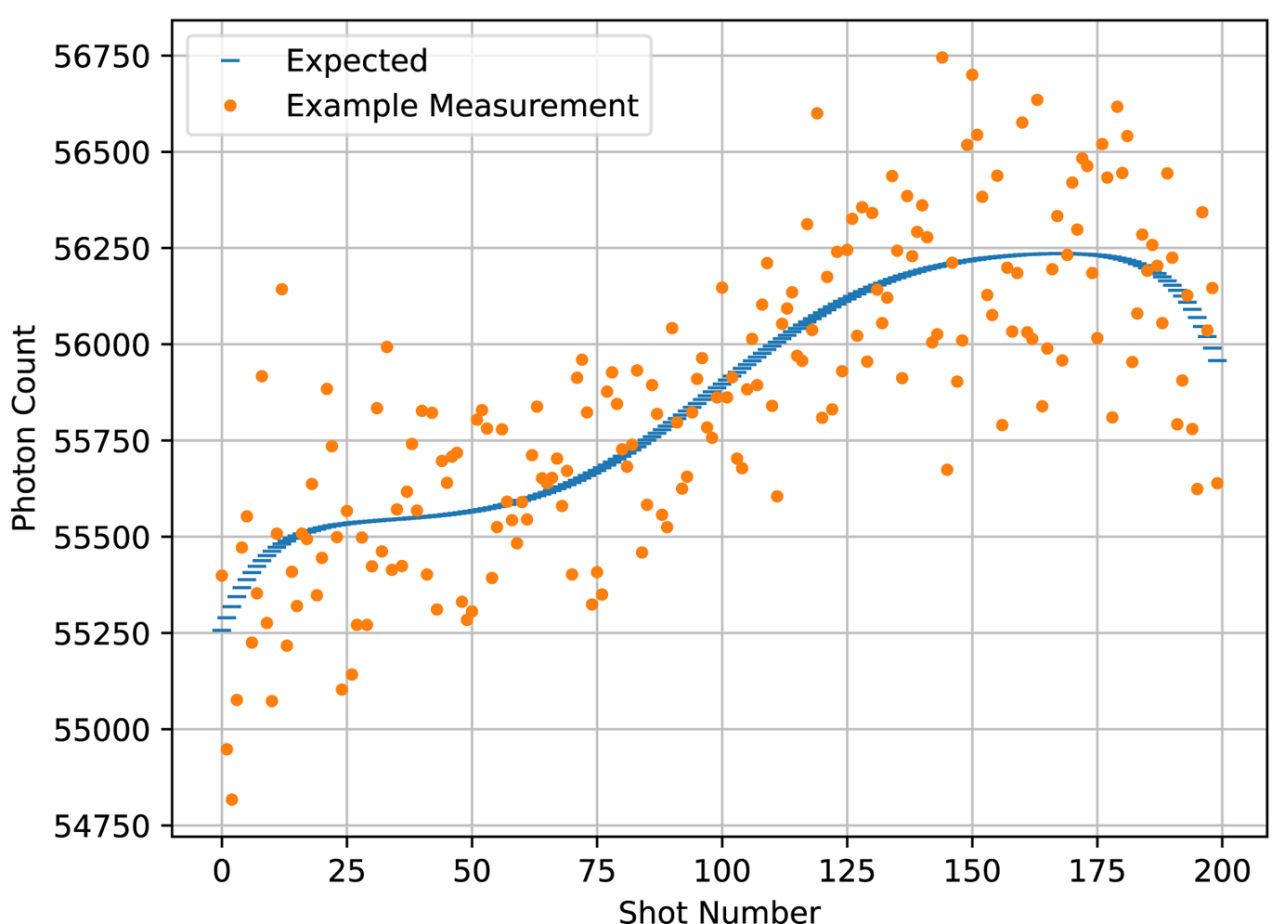
Pictured is an example of photon-count detections for each of the 200 time delays as detected by the 25th annular pixel. Each detection represents the sum of the photon counts incident on that pixel from 100 individual shots.

We chose our simulation parameters to roughly simulate the capabilities of the CXLS, that is, a probe beam diameter of 1 µm, a temporal pulse width 200 fs for both the pump and the probe, a temporal jitter of 50 fs, a probe pulse energy of 1 mJ, a probe photon energy of 9 keV or wavelength about 0.14 nm, a sample thickness of 5 µm and a sample concentration of 10 g l^−1^. For both temporal profiles, we simulated many repeated measurements at each of 200 time points. The temporal center of the beam is at −1.4 ps at the first time point, and 1.4 ps at the last, with the rest of the time points evenly spaced between. For the toy calmodulin temporal profile, we used an effective 20000 shots, either 1000 repetitions per time point in the individual SASE pulse case, or 100 repetitions, each representing the sum of 10 shots, in the binned Gaussian case. For the rhodopsin temporal profile, we used an effective 2000000 shots, a sum attainable at a typical beamtime, either 10000 repetitions in the SASE case or 100 repetitions each representing the sum of 100 shots in the binned Gaussian case. An example shot is shown in Fig. 5 ▸, and an example of a single annular pixel’s readout at each of the 200 time points is shown for the SASE pulses in Fig. 6 ▸ and for the binned Gaussian beam profile in Fig. 7 ▸.

## 4. Results

In the development of the algorithm, we assumed that the data are Gaussian-distributed about their expected values with the same variance for all data. We simulated the data using Poisson distributions, which have the property that their expected value and variance are the same. If the expected values of the data vary little over the time frame compared with their average magnitude, and that magnitude is large, then the Poissonian distributions can be well approximated with constant-width Gaussians by setting their variance to the mean of the data. These conditions are met in the case of the Gaussian beam profile data that represent the sum of many shots, as seen in Fig. 7 ▸. By contrast, in the single-shot SASE data as seen in Fig. 6 ▸, the expected value varies by an order of magnitude due to shot-wise variations in fluence, so the Gaussian distribution for the data is a much worse approximation. This mismatch creates a tension in the reconstruction that we wanted to test.

We estimated error bars on all of our reconstructions by running the algorithm on subsets of the data of just one datum for each measured time point, 100 subsets in the case of the many-shot Gaussian-limit data, and 1000 subsets in the case of the single-shot SASE data, and taking the point-wise standard deviation of the resulting reconstructions.

When plotting reconstructions, we only present the temporal profile of a single *q* bin. This is because the fictitious temporal scattering profiles were constructed such that the shape of the temporal profiles has the same essential features at every *q* value, so there is little benefit to displaying the reconstruction for every *q* bin. We chose to display the reconstruction for the 25th annular pixel at *q* = 1.3 Å^−1^ for all of our results.

**Figure 8:**
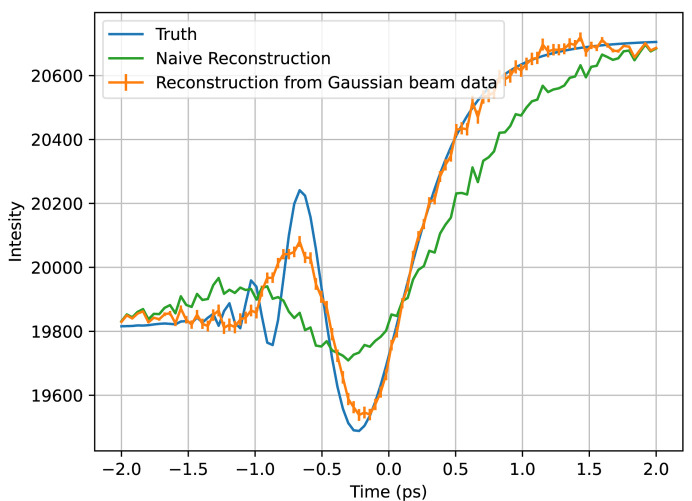
Plotted in orange is the algorithm’s reconstruction of the toy calmodulin temporal profile at *q* = 1.3 Å^−1^. The naive reconstruction is shown in green and the true temporal profile in blue. The naive reconstruction has a relative root mean square (RMS) error of 74%, our reconstruction has an error of 29%.

We first applied the algorithm to the toy calmodulin data sets. In the Gaussian beam profile case, the reconstruction, a single annular pixel of which is shown in Fig. 8 ▸, is accurate for the features of a similar scale and larger than of the combined beam temporal profile, but, with smaller features, it loses accuracy due to the lack of information present in our measurements of the temporal profile’s overlap with the shifted Gaussian beam profiles. This is due to the fact these Gaussians, though a linearly independent set of functions, are an inefficient basis with high overlap. It is still a great improvement in resolution compared with the naive reconstruction. At the edges of the time window, the algorithm’s reconstruction regresses to the naive reconstruction, an effect visible in all of the algorithm’s reconstructions. This is because the data set collected has almost no information about what happens at those time points, and the prior couples the algorithm’s reconstruction to the naive reconstruction; thus, in the absence of information from the data, the prior dictates the reconstruction should be as close to the naive reconstruction as possible.

**Figure 9:**
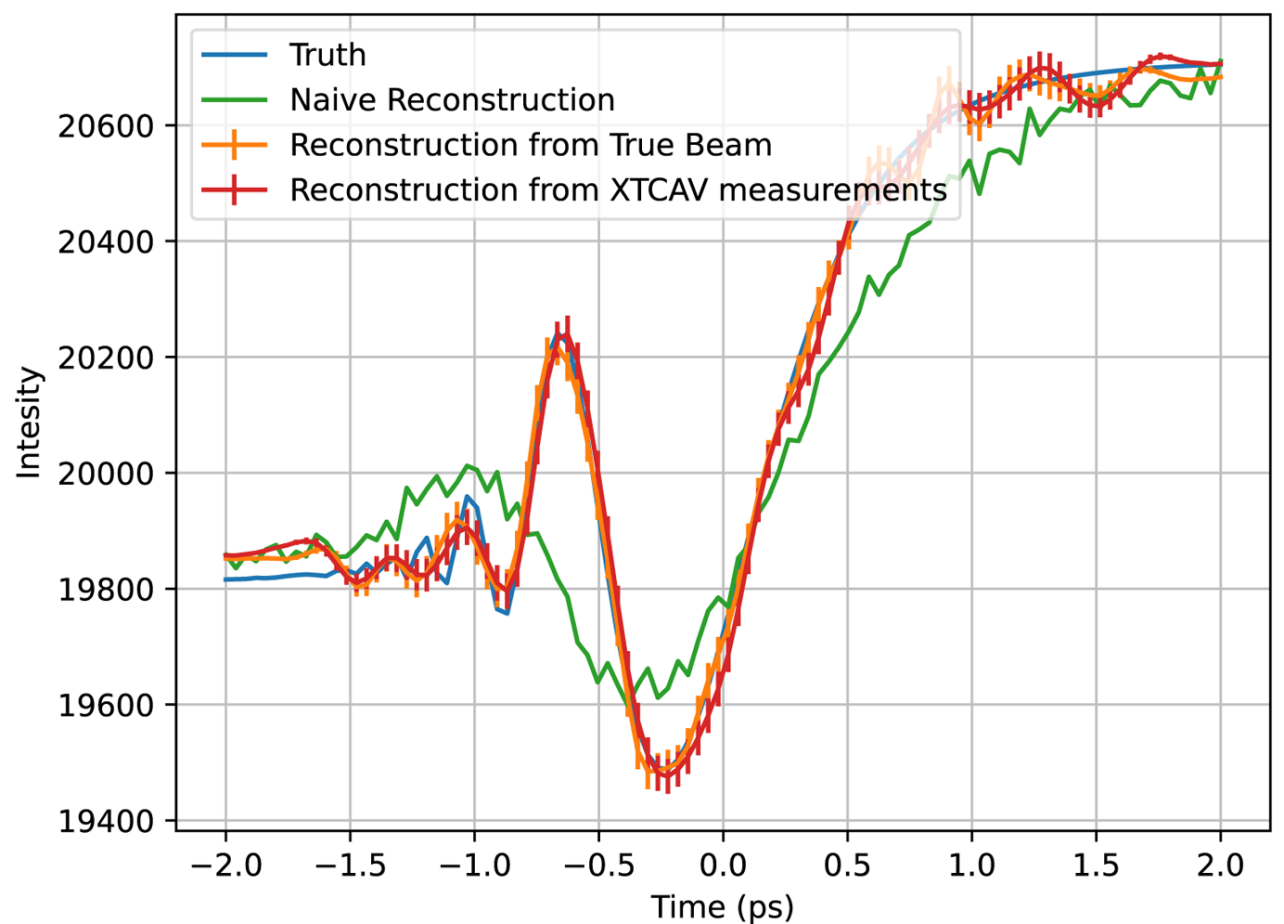
Plotted in orange is the algorithm’s reconstruction of the toy calmodulin temporal profile at *q* = 1.3 Å^−1^ from perfect beam measurements, in red is the reconstruction from the XTCAV data, in green is the naive reconstruction, and in blue is the true temporal profile. The naive reconstruction has a relative RMS error of 69%, the reconstruction from perfect beam measurements an error of 14%, and the reconstruction from the XTCAV beam measurements an error of 17%.

For the XTCAV data set, we ran the algorithm once with perfect knowledge of the beam, and once using our approximated XTCAV measurements, as exemplified in Fig. 3 ▸. The results for both are shown in Fig. 9 ▸. The temporal resolution is much higher for these reconstructions than the binned Gaussian result because the individual peaks in the beam profiles probe information at a temporal resolution smaller than the overall pulse duration, and their diversity due to random fluctuations provides a much higher information content. Artifacts are introduced, though, at the end of the time window, likely having to do with the aforementioned mismatch between the actual Poissonian statistics of the data and the Gaussian statistics assumed in the algorithm. The XTCAV reconstruction is only different by a small amount, demonstrating that a small perturbation in the beam matrix results in a small perturbation of the reconstruction.

**Figure 10:**
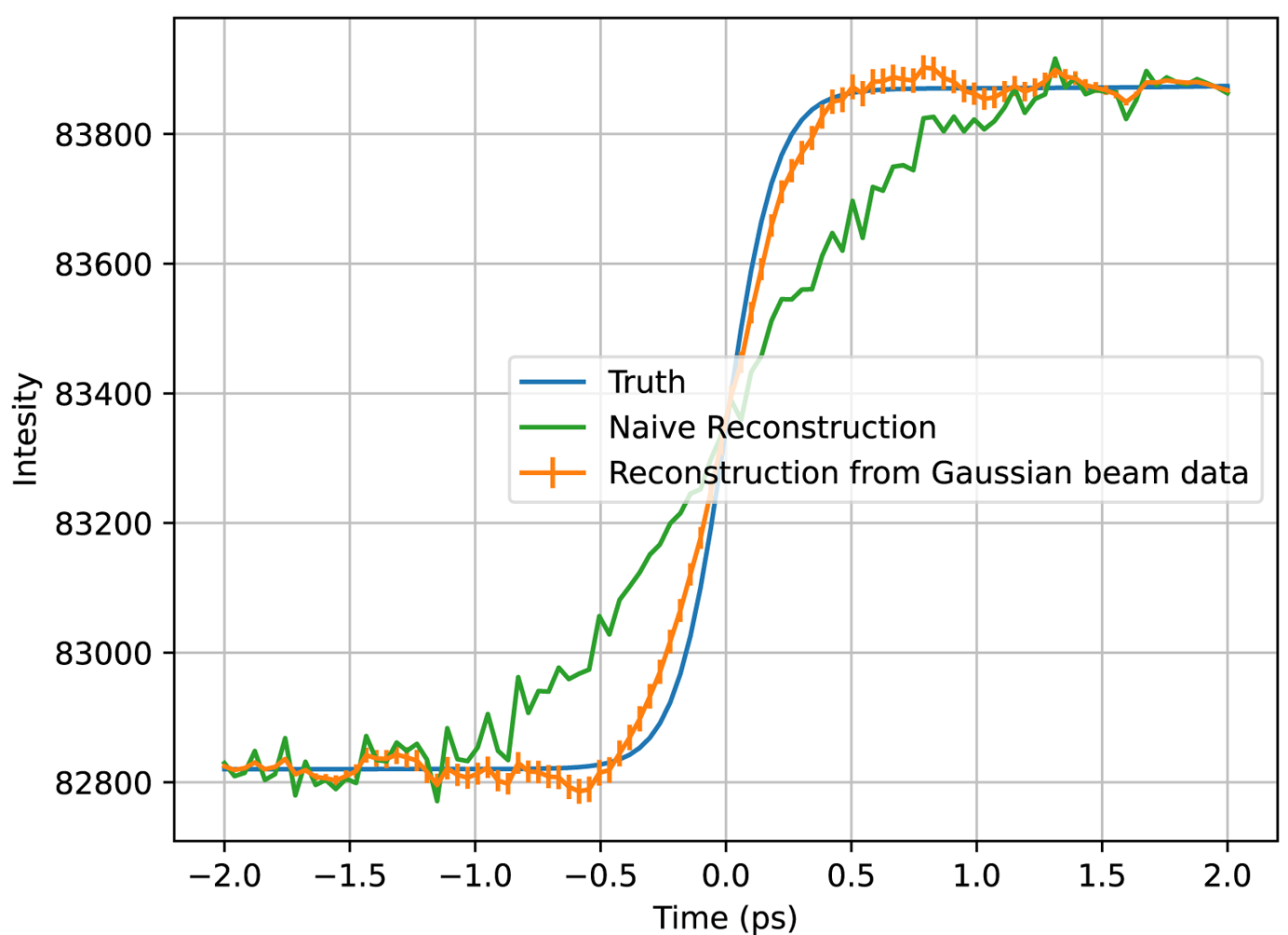
Pictured are the reconstructions of the temporal profile at *q* = 1.3 Å^−1^ from the binned Gaussian data set for rhodopsin. Plotted in orange is our reconstruction with error bars, in green is the naive reconstruction, and in blue is the true temporal profile. Our recovered timescale is 138 fs, the naively recovered timescale is 359 fs, and the true timescale is 100 fs.

We similarly applied the algorithm to the rhodopsin data sets, as shown in Figs. 10 ▸ and 11 ▸. We fitted our reconstructions to a logistic curve, which recovers the simulated photo-excitation timescale of the solution. The binned Gaussian data set recovered a timescale of 138 fs. For the SASE data set, the algorithm recovered a timescale of 88 fs with the perfect beam measurements and 93 fs with the XTCAV measurements. The naive reconstruction found a timescale of 359 and 272 fs, respectively, for the two data sets. The slight improvement of the XTCAV result over the perfect beam is presumably due to random fluctuations. It does indicate that, in this regime of small perturbations of the beam measurements, the discrepancy of counting statistics is the driving factor of error rather than the perturbations themselves. At higher levels of discrepancy between the true beam and measurements, the reconstruction degrades.

## 5. Discussion

The ghost imaging framework was successful in reconstructing super-resolution temporal features in our data set with a simple and fast computation. We have presented two modes that can be used, one of directly measuring individual shots, and one of binning shots and using only statistical beam measurements. We have shown that having shot-wise measurements of the beam, even ones that are slightly flawed, can significantly improve temporal resolution of the reconstructed evolution. In practice, these measurements may be too difficult to attain, especially in the exact timing of the delay between pump and probe pulse. The binned shot mode avoids this difficulty by averaging out shot-wise variation. It still achieves an improvement in resolution compared with the naive reconstruction, but inherently sacrifices resolution by losing information in the binning. One method to combat this timing uncertainty without throwing away useful information, but requiring far more computing power, is to use the framework of expectation maximization to maximize the likelihood or posterior marginalized over the possible pulse profiles. The approach requires a probabilistic model of the beam profiles, that is, a probability distribution over the space of all possible profiles. Any shot-wise or bulk measurements of the beam could be leveraged as prior information for this distribution. Another possible method would be to adapt the nonlinear Laplacian spectral analysis algorithm developed by Fung *et al.* (2016 ▸).

When using shot-wise measurements of the beam, our method leverages all of the information present in the measurement, including any fluctuations in the beam’s temporal profile. This stands in contrast to the much simpler method of improving temporal resolution by using the beam measurements to simply filter out the shortest pulses and restrict the analysis to the corresponding subset of the data (Liane *et al.*, 2024 ▸). Our method can achieve higher resolution, but is crucially dependent on the quality of the measurement of the beam, whereas the strategy of filtering out the shortest pulses conveniently only requires crude measurements.

Our algorithm reconstructs the evolution of the scattering profile of the entire solution, regardless of species present, for each *q* bin independently. In order to separate out contributions from different species in the mixture, one would need to employ an algorithm like REGALS (Meisburger *et al.*, 2021 ▸) on the output which leverages correlations between the *q* bins and various priors to identify the most likely time-evolving mixture. It may be possible to combine the two algorithms in a way that is more efficient than applying them serially.

We saw that, in the case of low-fluence data, the Gaussian approximation of the likelihood introduces artifacts into the reconstruction. In the extremely low fluence case where the approximation will break down entirely, it is possible to switch to a Poissonian formulation of the likelihood. The maximum likelihood solution is not analytic in this case, and will require numerical optimization. This will allow the reconstruction from sparse, photon-counting data sets, which may be helpful for lower-fluence sources like the CXLS.

## Figures and Tables

**Figure 11:**
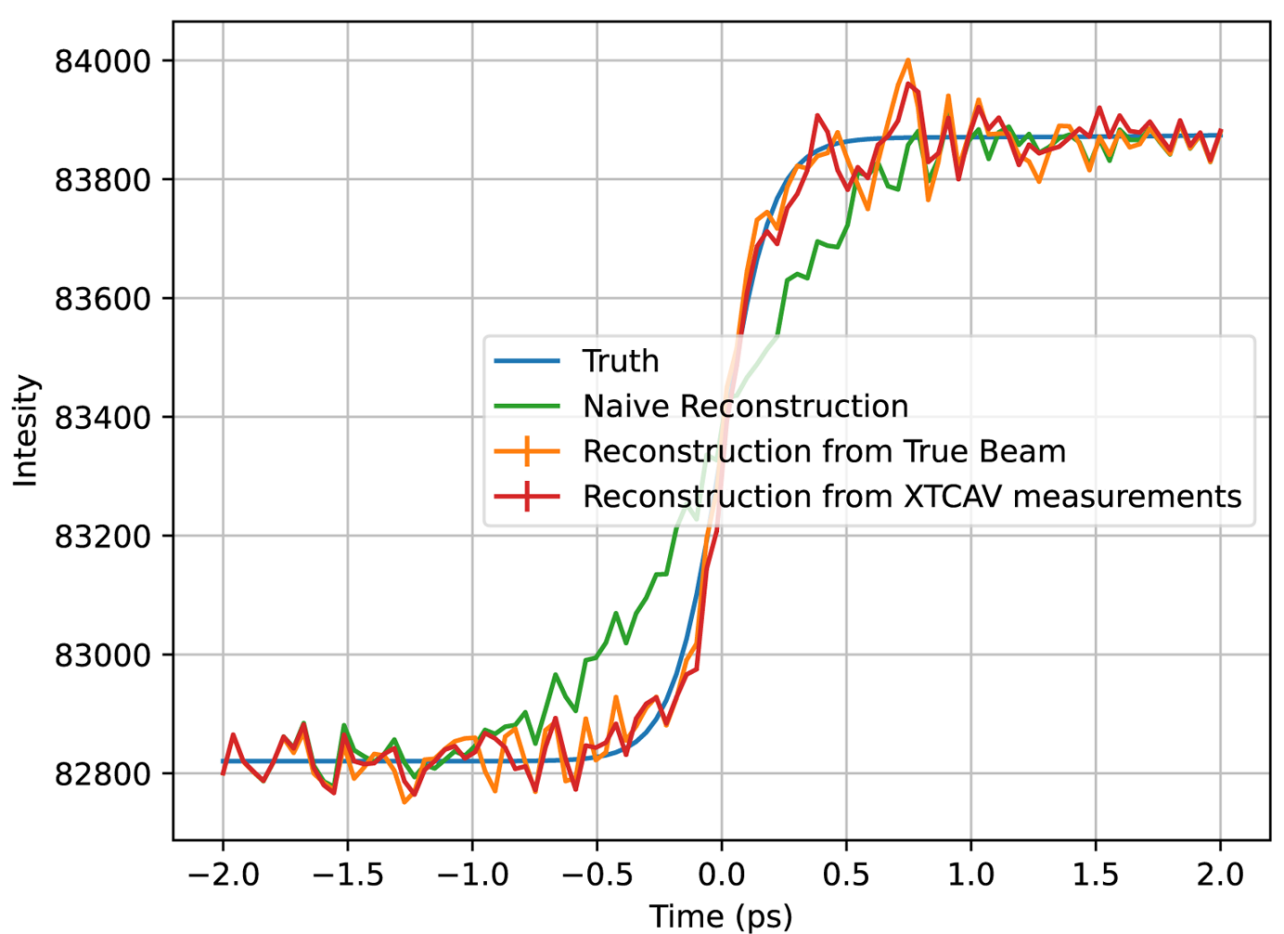
Plotted in orange is our reconstruction at *q* = 1.3 Å^−1^ from the perfect beam measurements, plotted in red is our reconstruction from the XTCAV beam measurements, plotted in green is the naive reconstruction, and plotted in blue is the true temporal profile for rhodopsin. The algorithm recovered a timescale of 88 fs and 93 fs for the perfect and XTCAV measurements, respectively. The naive timescale is 272 fs and the true timescale 100 fs. The error bars on the algorithm’s reconstructions are too small to see here.
